# Transdermal Administration of Volatile Oil from *Citrus aurantium*-*Rhizoma Atractylodis Macrocephalae* Alleviates Constipation in Rats by Altering Host Metabolome and Intestinal Microbiota Composition

**DOI:** 10.1155/2022/9965334

**Published:** 2022-01-18

**Authors:** Liangfeng Wang, Fang Wang, Xiaofei Zhang, Qingyao Chen, Jie Xu, Huiting Li, FengQin Li, Ming Yang

**Affiliations:** ^1^Key Laboratory of Modern Preparation of Traditional Chinese Medicine, Ministry of Education, Jiangxi University of Chinese Medicine, Nanchang 30004, China; ^2^College of Chinese Materia Medica, Shanghai University of Traditional Chinese Medicine, Shanghai 201203, China; ^3^College of Pharmacy, Shanxi University of Chinese Medicine, Shanxi 712046, China

## Abstract

**Background:**

The *Citrus aurantium*- (ZhiShi, ZS-) *Rhizoma Atractylodis Macrocephalae* (BaiZhu, BZ) pairs are often found in herbal formulas for constipation. The volatile oils of ZS and BZ (ZBVO) have good pharmacological activity against constipation, but the mechanism for treatment of slow transit constipation (STC) remains unclear.

**Method:**

A rat model using diphenoxylate tablets was constructed to investigate if transdermal administration of ZBVO would mediate intestinal microorganisms and fecal metabolites and improve STC symptoms. The regulatory effects of ZBVO at 0.15, 0.30, and 0.60 mL kg^−1^ d^−1^ on STC rats were assessed by measuring fecal water content, intestinal propulsion rate, histopathology, expression of gastrointestinal hormones, brain and intestinal peptides, and inflammatory factors. The changes in intestinal flora of STC rats were analyzed by 16S rRNA gene sequencing. Moreover, the untargeted fecal metabolomics analysis was performed by ultraperformance liquid chromatography quadrupole time-of-flight mass spectrometer (UPLC-Q-TOF-MS) technology.

**Results:**

The results showed that ZBVO had a modulating effect on STC by increasing the fecal water content and intestinal propulsion rate. Transdermal administration of ZBVO decreased serum levels of interleukin 6 (IL-6) and tumor necrosis factor-*α* (TNF-*α*) and increased the levels of gastrin (GAS) and substance P (SP). In addition, ZBVO increased 5-hydroxytryptamine (5-HT) levels and decreased vasoactive intestinal peptide (VIP) levels in colon and hippocampus tissues. The results of intestinal microbiota showed that ZBVO improved the diversity and abundance of intestinal microbiota and changed the community composition by decreasing *Romboutsia* and increasing *Proteobacteria*, *Allobaculum*, and *Ruminococcaceae*. And the feces metabolomics found that nicotinate and nicotinamide metabolism, purine metabolism, citrate cycle (TCA cycle), pyruvate metabolism, arachidonic acid metabolism, pyrimidine metabolism, and primary bile acid biosynthesis were modulated.

**Conclusion:**

These findings suggest that ZBVO can alleviate STC symptoms by promoting intestinal peristalsis, increasing fecal water content, regulating gastrointestinal hormone level, reducing the inflammatory response, and regulating brain and intestinal peptides after transdermal administration. And structural changes in the intestinal microbiota are closely related to host metabolism and intestinal microbiota destroyed in STC modeling could be significantly improved by the ZBVO, which provides a reference for the development of aromatic drug macrohealth products.

## 1. Introduction

Slow transit constipation (STC) is a common form of functional constipation characterized by reduced stool frequency, loss of bowel movement, dry and hard stools, and abdominal distension [1, 2]. Epidemiological studies indicate the global prevalence of STC is 14% [3], which seriously affects the physical and mental health of patients [4]. In addition, STC is closely associated with abdominal distension, cardiovascular and anal diseases, colorectal cancer, and Alzheimer's disease [5]. The etiology of STC is complex, and its mechanisms are not fully understood. The etiology of STC is mainly related to the enteric nervous system, gastrointestinal hormones, neurotransmitters, and intestinal microbiota [6, 7]. Currently, the main treatment methods for STC include medication and biofeedback therapy that often cause adverse reactions and drug dependence and highly invasive surgery that can have serious postoperative complications, such as abdominal pain and diarrhea [8, 9]. Therefore, natural medicines could offer better efficacy and applicability.

In most cases, a single drug does not completely cure STC; consequently, drug combinations are often used to exert synergistic effects and improve efficacy or reduce adverse side effects. STC has multiple causative factors. According to traditional Chinese medicine (TCM), the main cause is abnormal conduction of the large intestine, poor Qi flow, and internal stagnation of dregs and is closely related to the dysfunction of the spleen and stomach. In clinical practice, herbs that strengthen the spleen and regulate Qi are commonly used to treat STC. *Citrus aurantium*- (ZS-) *Rhizoma Atractylodis Macrocephalae* (BZ) is a classical drug pair that is recorded in many traditional formulas, such as Zhizhu decoction, and is commonly used to treat STC *Jinkui Yaolue*. ZS is a classical Qi-regulating herb that can treat stool obstruction and has the ability to break up Qi and eliminate stagnation and dissolve and disperse phlegm. ZS and its volatile oil have a long history of clinical application and are often used in TCM to treat stool obstruction, likely by enhancing the colon propulsion function and correcting the abnormal colonic slow waves of STC [10]. D-Limonene, the main component of ZS volatile oil, also has antibacterial activity [11]. BZ has the effect of replenishing Qi and tonifying the spleen, removing water from the intestinal track, and promoting water circulation. BZ volatile oil has a regulatory effect on gastrointestinal motility and body immunity, in addition to antibacterial, anti-inflammatory, and antiaging effects [12]. Its main active ingredient, atractylon, even has a laxative effect [13].

A transdermal drug delivery system not only avoids the first-pass effect but also is painless, aids patients with swallowing difficulties, increases patient compliance, and can be self-administered. The Shenque point means “Spirit Gate,” which is in the center of the abdomen and is a point that represents the final closure of the abdominal wall at the end of embryonic development. The Shenque point has a thin cuticle making it conducive to rapid drug absorption, and its location close to the intestinal tract strengthens the spleen and stomach by promoting the flow of Qi and relaxing the bowels [14]. This study takes the advantages of strong fast penetration and easy dispersal of Chinese medicine volatile oil, which can be absorbed through transdermal administration at the umbilicus, to adjust gastrointestinal dynamics, promote recovery of spleen and stomach functions, and achieve the therapeutic effect of moving Qi and laxative. However, the mechanism by transdermal administration of ZBVO improving STC remains unclear, therefore making it crucial to elucidate the basic mechanism of action in order to provide a new effective drug and treatment method for STC.

Intestinal microbiota has been implicated in the pathogenesis of various human diseases, such as irritable bowel syndrome (IBS), cardiovascular disease, and inflammatory bowel disease [15, 16]. Moreover, changes in intestinal microbiota composition are related to the pathogenesis of STC [17]. Dysbiosis of the intestinal microbiota may affect the brain-gut axis, thus promoting brain-gut peptide disorders. 16S rRNA gene sequencing studies enable accurate identification of the structure of intestinal microorganisms [18]. Metabolomics can comprehensively reflect the changes of metabolites in the process of disease occurrence and development [19]. Together, 16S rRNA gene sequencing and metabolomics have been widely used in the study of disease and drug treatment mechanisms, to reveal the pathogenic mechanism of diseases [20] and screening for biomarkers of diseases. However, the specific metabolites by which ZBVO affects host fecal metabolism via intestinal microbes are still unclear.

In the current study, we investigated the effects of ZBVO on the intestinal microbiota and fecal metabolite composition of STC rats induced by compound diphenoxylate tablets. To this end, we used 16S rRNA gene sequencing and ultraperformance liquid chromatography quadrupole time-of-flight mass spectrometer- (UPLC-Q-TOF-MS-) based metabolomics to determine the effects of ZBVO on the gut microbial community structure and fecal metabolomics of STC rats after transdermal administration via the umbilicus.

## 2. Materials and Methods

### 2.1. Materials and Chemicals

Compound diphenoxylate tablets (batch number: 1712029) were purchased from Changzhou Kangpu Pharmaceutical Co., Ltd. (Jiangsu, China). Mosapride (batch number: 190201) was purchased from Jiangsu Haosen Pharmaceutical Co., Ltd. (Jiangsu, China). The commercial enzyme-linked immunosorbent assay (ELISA) kits for tumor necrosis factor-*α* (TNF-*α*), interleukin 6 (IL-6), substance P (SP), gastrin (GAS), 5-hydroxytryptamine (5-HT), and vasoactive intestinal peptide (VIP) were purchased from Jiangsu Enzymatic Immunity Industry Co., Ltd. (Yancheng, China). Neutral gum and phosphate-buffered saline (PBS) solution were obtained from Beijing Solarbio Science & Technology Co., Ltd. (Beijing, China). HPLC-grade methanol, acetonitrile, isopropanol, ammonium hydroxide, and pure distilled water were obtained from Fisher Scientific (Fair Lawn, NJ, USA).

### 2.2. Plant Materials and Volatile Oil Extraction

*Citrus aurantium* (also known as ZhiShi in China) and *Rhizoma Atractylodis Macrocephalae* (also known as BaiZhu in China) were purchased from Chengdu Huichu Technology Co., Ltd. (Chengdu, China), which were authenticated by Professor Fei Ge (Jiangxi University of Traditional Chinese Medicine, Nanchang, China). According to the ratio of ZS : BZ = 2 : 1, the dried Chinese herbs were crushed into coarse powder. The herbs were distilled in seven volumes of water for 8 hours using an oil-water separator to produce ZBVO. The volatile oil samples were precisely absorbed from 100 *μ*L to 10 mL capacity flasks after anhydrous Na_2_SO_4_ dehydration, volume fixation with n-hexane, and passed through 0.22 *μ*m microporous filter membrane. Gas chromatography-mass spectrometry (GC-MS) was used to control the quality of ZBVO. The gas chromatographic conditions were as follows: Agilent DB-624 (30 m × 250 *μ*m, 0.25 *μ*m) capillary column, high-purity He (99.999%) carrier gas, and l *μ*L sample volume was used. The split ratio was nonshunt, and the flow rate was 1 mL/min. The temperature program was as follows: initial temperature of 40°C (held for 1 min), ramp to 10°C/min to 220°C, and then increased to 25°C/min to 280°C (held for 9 min). Mass spectrometry conditions were as follows: EI ion source, electron energy 70 eV, ion source temperature 230°C, MS quadrupole temperature 150°C, interface temperature 250°C, solvent delay 3.0 min, quality scan pattern full scan, and scan range of 30~650 amu. Standard spectral library NIST11 retrieval, peak area normalization method was used to calculate the relative percentage content of each component.

### 2.3. Animal Experimental Design

Sprague Dawley (SD) rats with a bodyweight of 200 ± 20 g were obtained from the Hunan Shrek Jingda Experimental Animal Co., Ltd. (Hunan, China; certificate number SCXK 2019-0004). The rats were housed at a standard laboratory environment (ambient temperature: 23 ± 1°C; 12 h light/dark cycle; relative humidity: 40%-70%) for one week with free access to food and water. All experimental procedures were approved by the Animal Ethics Committee of Jiangxi University of TCM and conformed to internationally accepted guidelines for the use of experimental animals.

**Figure 1:**
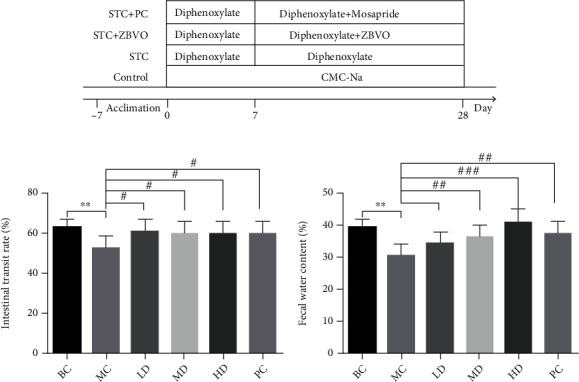
Effect of ZBVO on improving the fecal water content and intestinal transit rate of STC rats induced by compound diphenoxylate tablets (*n* = 8). (a) Schematic diagram of STC model establishment and transdermal drug administration treatment. (b) ZBVO improved intestinal propulsion rate and intestinal transit in diphenoxylate-induced STC rats (150, 300, and 600 *μ*L kg^−1^ d^−1^) and mosapride. (c) Compared with the MC group, ZBVO dosing (150, 300, and 600 *μ*L kg^−1^ d^−1^) increased fecal water content in rats. Values are expressed as mean ± SEM. ^∗^*p* < 0.05, ^∗∗^*p* < 0.01, and ^∗∗∗^*p* < 0.001 vs. the BC group. ^#^*p* < 0.05, ^##^*p* < 0.01, and ^###^*p* < 0.001 vs. the MC group.

After a week of adaptive feeding, the rats (24 male and 24 female) were randomly divided into six groups (four male and four female rats in each group): blank control (BC) group, model control (MC) group, low-dose (LD) treatment group, middle-dose (MD) treatment group, high-dose (HD) treatment group, and positive control (PC) group. The diphenoxylate-induced mouse STC model was established as previously described, with minor modifications [21]. After acclimation to the conditions for 1 week, except for the BC group, rats were treated with diphenoxylate (10 mL/kg) solution soluble in 0.5% sodium carboxymethyl cellulose (CMC-Na) by oral gavage once a day for 28 days. Starting on the seventh day, the LD, MD, and HD groups had the appropriate dosage (0.15, 0.3, and 0.6 mL kg^−1^ d^−1^, respectively) diluted with jojoba oil at 10 times the dosage, smeared on the Shenque point (the Shenque point is located one-third of the way down from the line connecting the symphysis of the rat's saber and pubic bone) for 21 days. Rats in the PC group were given mosapride (1.5 mg kg^−1^ d^−1^) solution soluble in 0.5% CMC-Na by intragastric administration once a day for 21 days. The rats in the BC group were treated with the same volume of CMC-Na solution by oral gavage once a day for 28 days (see Figure 1(a)).

### 2.4. Sample Collection

At the end of the intervention, on the 28^th^ day, 2 hours after the final administration, fresh fecal samples were collected and stored at -80°C. DNA was immediately extracted from fecal samples for microbiome analysis. All rats were fasted overnight (12 h) and then given 2 mL activated carbon suspension by intragastric administration. Forty minutes later, they were lightly anesthetized by intravenous injection of 1% pentobarbital (40 mg pentobarbital/kg rat bodyweight) and blood was collected from the abdominal aorta. The blood was centrifuged at 3000 rpm for 10 min at 4°C to obtain serum. The sera were stored at -80°C until analyzed. The small intestines, from pylorus to lower caecum, were extracted from every rat. At the same time, the colon tissue was cut open quickly; part of the colon was frozen in liquid nitrogen and then preserved at -80°C, while the remaining portion was fixed in 4% paraformaldehyde for further study.

### 2.5. Fecal Water Content and Intestinal Propulsion Rate

In this study, the water content in rat feces was calculated as described earlier [22]. In short, the feces of rats in each group were collected and dried at 50°C oven for 4 hours, and the fecal water content was calculated as follows:

(1) $$\begin{array}{c} \text{fecal water content}=\left(\frac{\text{wet weight}-\text{dry weight}}{\text{wet weight}}\right)\times 100\%. \end{array}$$

A scrape from the small intestine (the front end of the cecum) was placed on a clean glass plate without traction. We measured the total length and the distance from the front end of the carbon powder to the pylorus to calculate the ratio to the total length:

(2) $$\begin{array}{c} \text{intestinal propulstion rate}=\left(\frac{\text{charcoal powder propulsion distance }\left(\text{cm}\right)}{\text{intestinal full length }\left(\text{cm}\right)}\right)\times 100\%. \end{array}$$

### 2.6. Determination of Gastrointestinal Hormones (GAS and SP) and Inflammatory Factors (TNF-*α* and IL-6)

The serum was taken and the contents of GAS, SP, IL-6, and TNF-*α* were determined by using ELISA kits accordingly, and the absorbance was set at 450 nm and measured on a microplate reader.

### 2.7. Determination of Brain-Gut Peptide (5-HT and VIP)

Colon and hippocampal tissues were homogenized with PBS at a ratio of 1 : 9 (weight : volume) on ice using a glass homogenizer, respectively. The supernatant was obtained by centrifugation at 3500 rpm/min at 4°C for 10 min. The total protein in the sample was detected with a BCA kit, and the content of 5-HT and VIP in the colon and hippocampal tissues was determined by using ELISA kits accordingly. The 5-HT and VIP contents were measured at 450 nm on a microplate reader. Finally, the fruit value was calculated as

(3) $$\begin{array}{c} \text{fruit value}=\frac{\text{ELISA concentration value}}{\text{BCA concentration value}}. \end{array}$$

### 2.8. Pathological Evaluation

The rat colon was fixed in 4% paraformaldehyde for 24 hours, routinely dehydrated, embedded, sectioned, and stained with hematoxylin-eosin stain according to the standard experimental procedure. The pathological histological changes were observed under a light microscope with a ×100 objective.

### 2.9. 16S rRNA Gene-Based Bacterial Community Analysis

Community structure of rat intestinal microbiota was analyzed by 16S rRNA gene sequencing technique. A genomic DNA isolation kit (MoBio Laboratories, Carlsbad, CA) was used to extract total DNA samples from the rat feces samples. The extracted DNA was detected by 1% agarose gel electrophoresis, and quality and concentration were determined using spectrophotometry. Paired-end sequencing was performed using the Illumina MiSeq PE300 high-throughput sequencing platform at Beijing Aoweisen Gene Technology Co., Ltd. The primers 515F (5′-GTGCCAGCMGCCGCGGTAA-3′) and 806R (5′-GGACTACHVGGGTWTCTAAT-3′) were used to amplify the bacterial 16S rRNA gene V4 region. The PCR products were detected by 1% agarose gel electrophoresis for amplification target band size and purified with an Agencourt AMPure XP Nucleic Acid Purification Kit. The raw sequences were uploaded to NCBI's SRA database. Pairs of reads were spliced into one sequence, and the data were optimized for OTU (97% similarity level) clustering analysis using the RDP Classifier algorithm. Diversity analysis and multilevel discriminate analysis size effect (LEfSe) were performed on all samples; environmental factor correlation analysis was performed using the Spearman correlation analysis.

### 2.10. UPLC-Q-TOF-MS Analysis

A 30 mg rat fecal sample was placed into a 2 mL EP tube, and then, 500 *μ*L of extraction solution was added (methanol : acetonitrile : water = 2 : 2 : 1). Samples were processed using a 60 Hz grinder for 2 min, sonicated in an ice water bath for 10 min, and kept at -20°C for 1 h. Samples were centrifuged at 13,000 r/min for 15 min at 4°C, and then, 350 *μ*L of supernatant was aspirated and placed in a vacuum freeze dryer until evaporated and dry. Then, 100 *μ*L of extraction solution (acetonitrile : water = 1 : 1) was added to the dried metabolites for resolubilization and vortexed for 30 s, followed by 10 min of sonication in an ice-water bath. We then centrifuged at 13,000 r/min for another 15 min at 4°C. Next, we placed 50 *μ*L of the resulting supernatant into the injection bottle and waited for UPLC-Q-TOF-MS detection. The samples were separated in Waters Acquity UPLC BEH Amide (2.1 × 100 mm, 1.7 *μ*m). The mobile phase consisted of (A) ultrapure water (containing 25 mM ammonium acetate and 25 mM ammonium hydroxide) and (B) acetonitrile. The elution gradient is shown in Supplemental Table 1. The flow rate was 0.5 mL/min, column temperature was 40°C, and the injection volume was 2 mL. Briefly, the mass spectrometry conditions were set as follows: electrospray ion source temperature (TEM) of 650°C; mass spectrometry voltage (ISVF) 5500 V (positive ions), -4500 V (negative ions); declustering voltage (DP) 60 V; ion source gas 60 psi for both gas 1 and gas 2, gas curtain (CUR) 30 psi; atomization pressure (GS1) 60 Pa, auxiliary pressure 60 Pa, spray voltage 5500 V (positive ion mode) or -4500 V (negative ion mode), mass spectrometry scan range MS1 60-1200 Da, MS2 25-1200 Da.

### 2.11. Statistical Analysis

Statistical analyses were performed using IBM SPSS Statistics 21.0 (Chicago, USA). The experimental data in this study are presented as mean ± standard deviation (SD). Single-factor ANOVA was used for analysis, LSD method was used for homogeneity of variance, Games-Howell method was used for heterogeneity of variance, and *p* < 0.05 was considered a significant difference and *p* < 0.01 was considered a very significant difference.

MarkerView 1.3.1 software was used for peak identification, peak extraction, and peak alignment, while minfrac and cutoff were set to 0.5 and 0.6, respectively. Substance identification of peaks was performed by secondary mass spectrometry database, R program package. The missing values that appear in the original data are simulated, and the simulation method is the minimum value 1 : 2 method for filling. The low-quality ions were removed according to the “80 % principle,” and finally, the peak area was normalized using the total ion flow of each sample. Univariate Student's *t*-test between the blank group and the STC model group was performed, and statistical significance was considered as *p* < 0.05. Multivariate analyses, including unsupervised PCA and supervised OPLS-DA analysis, were performed using SIMCA 14.1 software, and the model was validated. Statistical significance was considered as *p* < 0.05 in the *t*-test, and VIP ≥ 1 was used to screen for difference variables. Human metabolome database (HMDB, http://www.hmdb.ca) and the Kyoto Encyclopedia of genes and genomes (KEGG, http://www.kegg.jp) database were selected for metabolite identification, and the identified differential metabolites were analyzed by the Pathway Analysis module in the MetaboAnalyst 3.0 platform for metabolic pathway analysis with the pathway impact > 0 and −log(*p*) > 0.5 set as screening conditions.

## 3. Results

### 3.1. Composition Analysis of ZBVO

**Figure 2:**
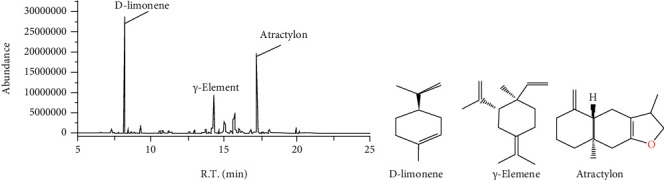
GC-MS analysis of ZBVO. (a) GC-MS analysis of the TIC component of ZBVO. (b) Structures of three main chemical compounds isolated from ZBVO.

The main chemical composition and relative percentage of ZBVO analyzed by GC-MS are shown in Table 1. A total of 38 chemical components were identified, with the majority being D-limonene (42.72%), followed by atractylon (27.51%), and then *γ*-elemene (7.83%). GC-MS analysis of the Total Ion Chromatography (TIC) component of ZBVO is shown in Figure 2(a). Structures of D-limonene, atractylon, and *γ*-elemene are presented in Figure 2(b).

### 3.2. Effects of ZBVO on Intestinal Motility and Fecal Water Content

Figure 1 shows the effects of ZBVO on intestinal motility and fecal water content in STC rats. In Figure 1(b), the MC group had significantly lower intestinal motility velocity than the BC group (*p* < 0.01), indicating that the STC model was successfully induced. All three doses of ZBVO and mosapride were effective in promoting intestinal motility in STC rats (*p* < 0.05). In Figure 1(c), the MC group showed a significant decrease in fecal water content compared to the BC group (*p* < 0.001), while ZBVO transdermal administration showed a dose-dependent increase in fecal water content (*p* < 0.01). These results suggest that ZBVO treatment effectively promoted defecation and enhanced colonic dynamics in rats.

### 3.3. Effects of ZBVO on Gastrointestinal Hormones (GAS and SP) in the Serum, Pathological Histology of Colonic Tissue, and Inflammatory Factors (TNF-*α* and IL-6) in the Serum

**Figure 3:**
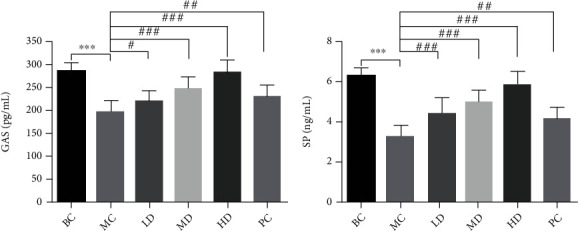
Effects of ZBVO treatment on (a) GAS and (b) SP levels in the serum (*n* = 8). Compared with the normal group: ^∗∗^*p* < 0.01 and ^∗^*p* < 0.05; compared with the model group: ^##^*p* < 0.01 and ^#^*p* < 0.05.

**Figure 4:**
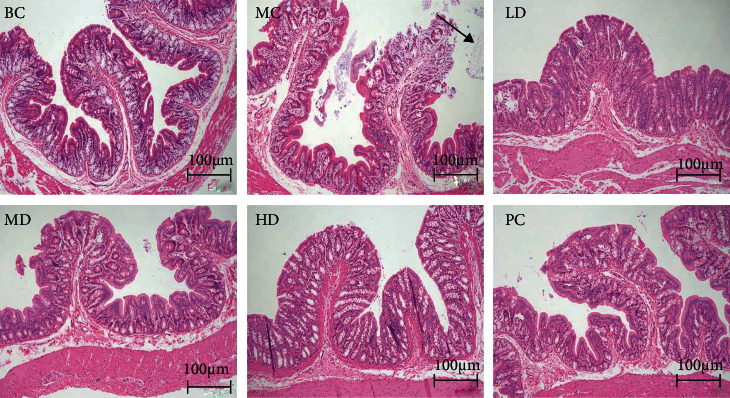
Histopathology examination of the colon in STC rats. H&E staining of the colon (*n* = 3).

**Figure 5:**
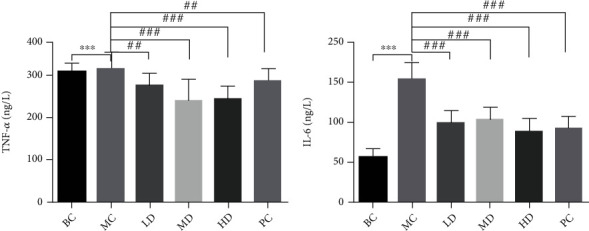
Effect of ZBVO on the content of IL-6 and TNF-*α* in the serum (*n* = 8). Values are expressed as mean ± SEM. ^∗^*p* < 0.05, ^∗∗^*p* < 0.01, and ^∗∗∗^*p* < 0.001 vs. the BC group. ^#^*p* < 0.05, ^##^*p* < 0.01, and ^###^*p* < 0.001 vs. the MC group.

Assessment of serum levels of gastrointestinal hormones and inflammatory factors can be used to reveal the role of ZBVO in STC regulation. GAS and SP levels in the MC group were significantly lower than those in the BC group (*p* < 0.001) (Figures 3(a) and 3(b)). However, transdermal administration of ZBVO in the PC group was effective in upregulating serum levels of GAS and SP compared to that in the MC group, and the effect of ZBVO was dose-dependent, with the best effect in the HD group. H&E staining of colon tissue sections showed that no significant morphological damage was observed. Breakage and loss of epithelial cells in the mucosal layer of colonic tissue and reduction of cupped cells were observed in colon tissue sections from rats with STC induced by diphenoxylate tablets. However, ZBVO and mosapride treatment improved the colonic mucosal injury (Figure 4). The levels of relevant inflammatory factors in serum are shown in Figures 5(a) and 5(b). The levels of IL-6 and TNF-*α* in the MC group were significantly higher than those in the BC group (*p* < 0.001), and the levels of IL-6 and TNF-*α* were reduced after ZBVO and mosapride treatment, and the levels of IL-6 and TNF-*α* in ZBVO-HD group were more similar to those in the BC group. The results showed that ZBVO had a dose-dependent modulating effect on STC with ZBVO-HD having the best effect.

### 3.4. Effects of ZBVO on Brain Intestinal Peptide (5-HT and VIP)

**Figure 6:**
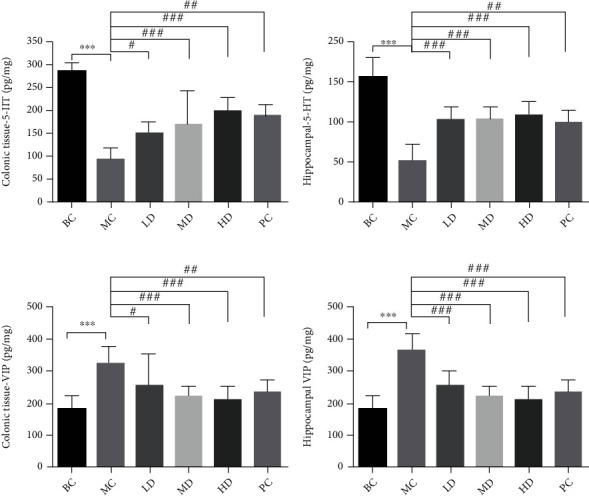
(a–d) Effects of ZBVO treatment on 5-HT and VIP levels in colon and hippocampal tissues (*n* = 8). Values are expressed as mean ± SD, *n* = 8. ^∗^*p* < 0.05, ^∗∗^*p* < 0.01, and ^∗∗∗^*p* < 0.001 vs. the BC group. ^#^*p* < 0.05, ^##^*p* < 0.01, and ^###^*p* < 0.001 vs. the MC group.

In order to observe the regulatory effect of ZBVO on the gut-brain axis in STC rats, the levels of 5-HT and VIP in colon and hippocampal tissues were determined by ELISA. As shown in Figures 6(a) and 6(b), the 5-HT content in the colon and hippocampal tissues of the STC model group was lower than that of the BC group (*p* < 0.001). Compared with that of the MC group, the 5-HT content in the colon and hippocampal tissues of the ZBVO-LD, ZBVO-MD, ZBVO-HD, and PC groups was significantly higher (*p* < 0.05 or *p* < 0.01 or *p* < 0.001), indicating that ZBVO promoted the secretion of 5-HT in the colon and hippocampal tissues. In Figures 6(c) and 6(d), compared with that in the BC group, the VIP content in the colon and hippocampal tissues was decreased in the MC group (*p* < 0.001); compared with the MC group, the ZBVO-LD, ZBVO-MD, ZBVO-HD, and PC groups all decreased the VIP content in the colon and hippocampal tissues to different degrees (*p* < 0.05 or *p* < 0.01 or *p* < 0.001).

### 3.5. Regulation of Intestinal Microbiota in Diphenoxylate-Induced STC Rats

**Figure 7:**
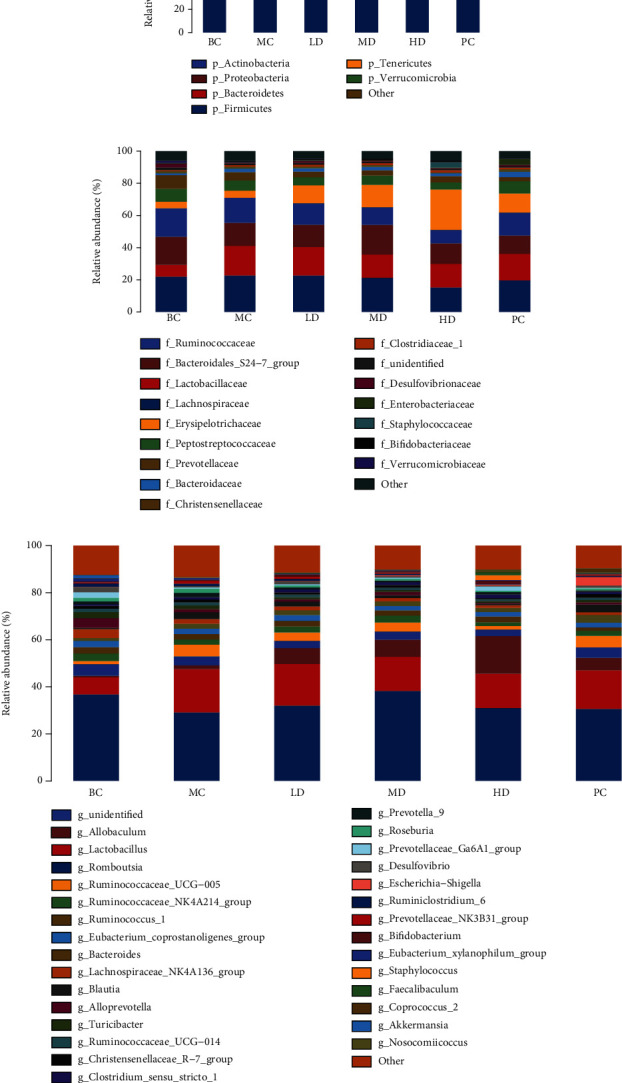
Analysis of fecal microbial composition of rats in each group: (a) phylum level, (b) family level, and (c) genus level. Less than 1% of the species were classified as “other” species at all taxonomic levels.

Thirty-six samples from six groups were evaluated. In order to ensure high coverage for the samples, the data volume of all samples was homogenized to 26,251 sequences. As shown in Supplemental Figure 1A, the rank-abundance curve tends to be smoother, indicating the more uniform distribution of species. As shown in Supplemental Figure 1B, the sample curves were all flat and concentrated, indicating that the sequencing data were large enough to reflect the majority of microbial information in the samples. The analysis of the microbiota community structure at the phylum level is shown in Figure 7(a), and the results indicate that the identified microorganisms were mainly *Firmicutes*, *Bacteroidetes*, *Proteobacteria*, and *Actinobacteria*, with *Firmicutes* accounting for the largest proportion. Compared to the BC group, diphenoxylate-induced STC rats resulted in higher abundance of *Firmicutes* (72.95% vs. 64.13%); *Bacteroidetes* (22.12% vs. 28.01%) and *Proteobacteria* (1.48% vs. 3.20%) were less abundant than those in the BC group. Compared with the MC group, the abundance of *Firmicutes* decreased in the MD group and increased in the LD, HD, and PC groups. The abundance of *Bacteroidetes* decreased in the LD, HD, and PC groups but increased in the MD group. The relative abundance of *Proteobacteria* increased in the LD, MD, HD, and PC groups. The analysis of the microbiota community structure at the family level is shown in Figure 7(b). Diphenoxylate-induced STC rats showed higher family of *Ruminococcaceae*, *Lactobacillaceae*, and *Erysipelotrichaceae*, but lower *Lachnospiraceae*, *Bacteroidales*_S24-7_group, *Prevotellaceae*, and *Peptostreptococcaceae* families. At the genus level, we found that the relative abundance of *Lactobacillus*, *Ruminococcaceae*_UCG-005, and *Allobaculum* was significantly increased in the STC group compared with the BC group, while the relative abundance of *Romboutsia* was significantly decreased. Compared with the MC group, the LD, MD, HD, and PC groups increased the relative abundance of *Allobaculum*, and the LD, MD, and HD groups decreased the relative abundance of *Romboutsia* and increased the relative abundance of *Ruminococcaceae*_UCG-005, while the PC group showed the opposite.

**Figure 8:**
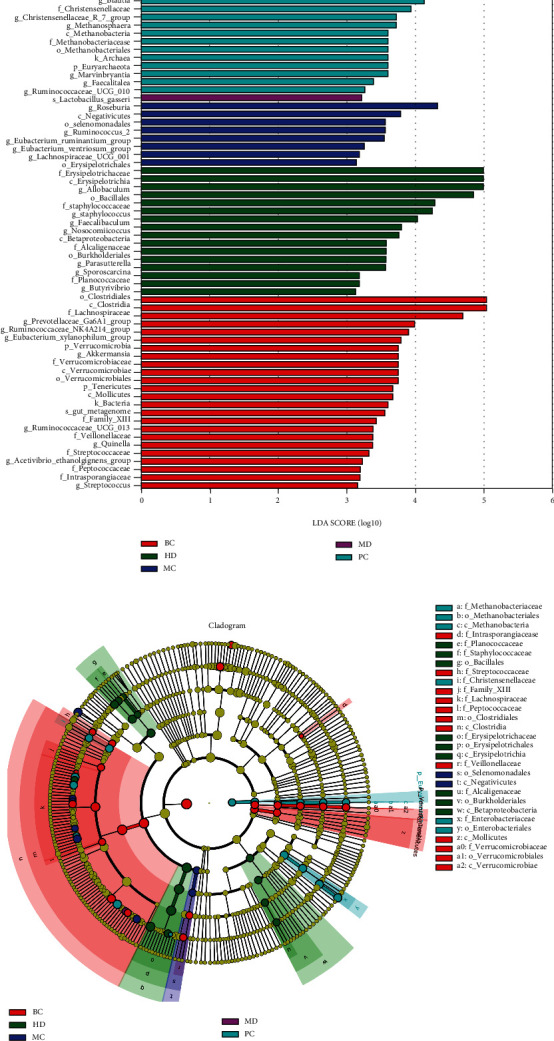
Effect of ZBVO on gut microbiota abundance in STC rats. (a) Distribution histogram based on LDA. (b) Bar plot analysis of community abundance on the genus level.

To identify bacterial taxa that differed significantly between groups, LEfSe (linear discriminate analysis size effect) (values > 3) analysis was performed between groups (Figure 8). From phylum to genus, a total of 66 taxa were obtained from all groups. Among them, the BC group was enriched to 24 taxa, the MC group to 8 taxa, the MD group to one taxon, the HD group to 16 taxa, and the PC group to 17 taxa, while the LD group was not enriched to taxa. The results showed that o__*Clostridiales*, c__*Clostridia*, and f__*Lachnospiraceae* were more abundant in the BC group; s__*Lactobacillus_gasseri* and g__*Roseburia* were more abundant in the MC group, while only g__*Ruminococcaceae*_UCG_010 was screened in the LD group at values > 3. In the HD group, the abundance of c__*Erysipelotrichia*, f__*Erysipelotrichaceae*, o__*Erysipelotrichales*, and o__*Bacillales* was higher, while in the PC group g__*Ruminococcaceae*_UCG_005, s__*Escherichia*_*coli*, g__*Escherichia*_*Shigella*, etc. were more abundant.

**Figure 9:**
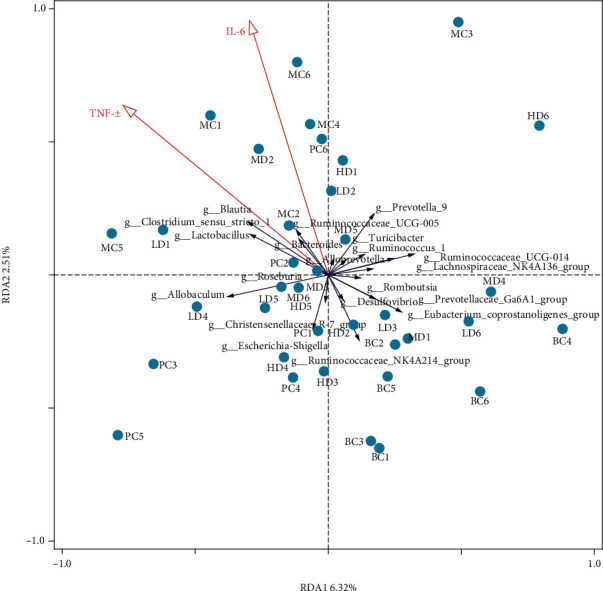
Analysis of RDA correlation between inflammatory factors and intestinal microorganism genus level where the dots represent the 36 samples (*n* = 6). Red rays represent the two environmental factors, IL-6 and TNF-*α*, and blue rays represent different species. The length of the rays represents the degree of their influence on the sample communities, and the direction of the rays represents the direction of increasing abundance. If the angle between the two rays is acute, it means there is a positive correlation; if it is obtuse, then there is a negative correlation; and if it is right angle, there is no correlation.

The redundancy analysis (RDA) was used to investigate the correlation between the serum levels of host environmental factors IL-6 and TNF-*α* and intestinal microbiota in each group of rats. The percentages of the horizontal and vertical coordinates indicate the weight of explanation for the differences in sample composition. The results are shown in Figure 9. Combined RDA and Spearman correlation results show that *Lactobacillus* and *Blautia* showed a weak positive correlation to TNF-*α* (*R* > 0.3, *p* < 0.05), *Ruminococcaceae*_NK4A214_group showed a negative correlation with IL-6 and TNF-*α* (*R* = −0.39, *p* < 0.05, *R* = −0.36, *p* < 0.05), and *Blautia* showed a positive correlation with IL-6 (*R* = 0.37, *p* < 0.05); a significant positive correlation was also found between the two environmental factors (*R* = 0.84, *p* < 0.001).

### 3.6. Regulation of Fecal Metabolomics in Diphenoxylate-Induced STC Rats

**Figure 10:**
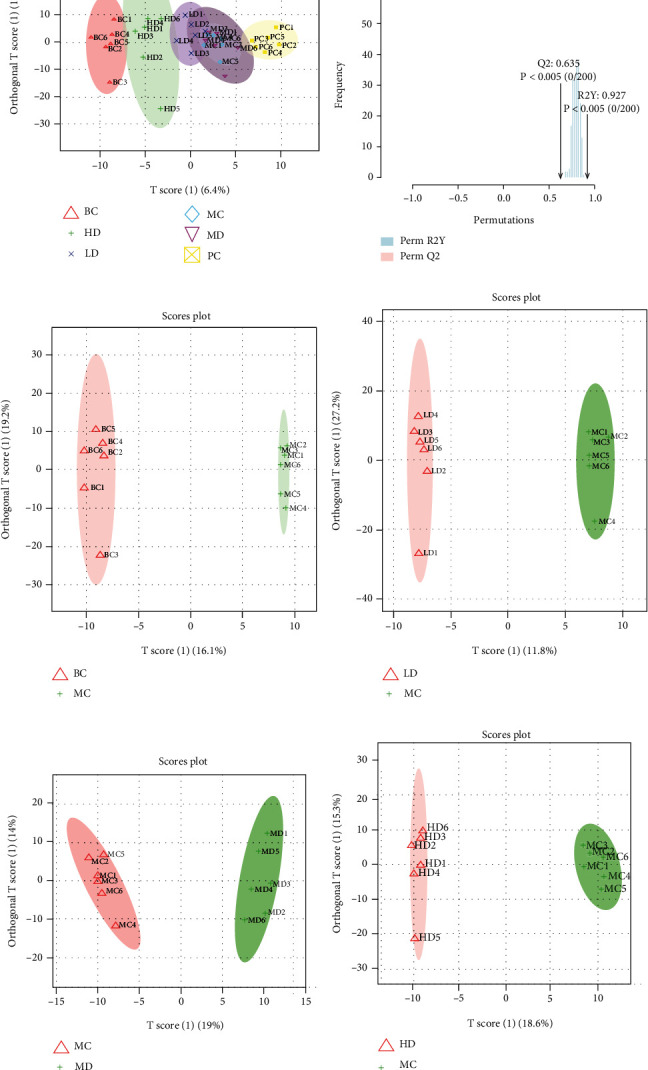
Supervised discriminant analysis of fecal metabolome in STC rats (*n* = 6). (a) OPLS-DA analysis and (b) validation of six groups. OPLS-DA analysis of (c) BC vs. MC, (d) LD vs. MC, (e) MD vs. MC, and (f) HD vs. MC.

The positive and negative ion metabolic profiles of each group of stool samples were analyzed by UPLC-Q-TOF-MS; these results were combined and analyzed to establish orthogonal partial least squares discrimination analysis (OPLS-DA) (Figure 10). The separation between the MC and BC groups was clearly shown in both OPLS-DA analyses (Figure 10(a)), indicating that the metabolic phenotype of STC rats has changed significantly. The fecal metabolic profile of the LD, MD, and HD groups, with the HD group being the most obvious, changed similarly to the BC group and showed partial overlap, indicating that the high dose of ZBVO had a significant ameliorating effect on STC rats. In addition, in the OPLS-DA model validation, the model parameters *R*^2^*Y* = 0.927 and *Q*^2^ = 0.635, *p* < 0.005, indicate that the model has good adaptability (Figure 10(b)). OPLS-DA analysis provides better separation and can be used to further search for biomarkers (Figures 10(c)–10(f)).

The HMDB online database and KEGG database were used to screen metabolites using *p* < 0.05 and VIP ≥ 1 parameters. Thirty significantly changed metabolites were identified in feces (Table 2), 25 were significantly lower in the MC group than in the BC group, and five were significantly upregulated in the STC rats. After ZBVO treatment, some metabolites did not show significant changes in the diphenoxylate tablet-induced model group compared to the treatment group. However, uridine, glycochenodeoxycholate, arachidonic acid (peroxide-free), quinolinate, 1-methylnicotinamide, xanthosine, 3-phenylpropanoic acid, azelaic acid, L-malic acid, and deoxyadenosine showed a significant inverse trend (Table 3). This suggests that the mechanism of action of ZBVO in regulating STC may be related to these metabolites, likely by interfering with different metabolic pathways.

**Figure 11:**
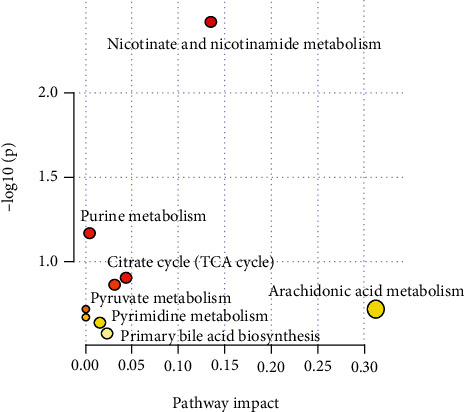
Summary of pathway analysis with MetPA.

To investigate the potential metabolic pathways of ZBVO in STC, the 10 metabolites with significant differences in Table 3 were subjected to metabolic pathway analysis and the results showed that ZBVO regulates seven metabolic pathways in STC rats (Figure 11), namely, nicotinate and nicotinamide metabolism, purine metabolism, citrate cycle (TCA cycle), pyruvate metabolism, arachidonic acid metabolism, pyrimidine metabolism, and primary bile acid biosynthesis.

### 3.7. Relevance Analysis between Gut Microbiota and Fecal Biomarkers

**Figure 12:**
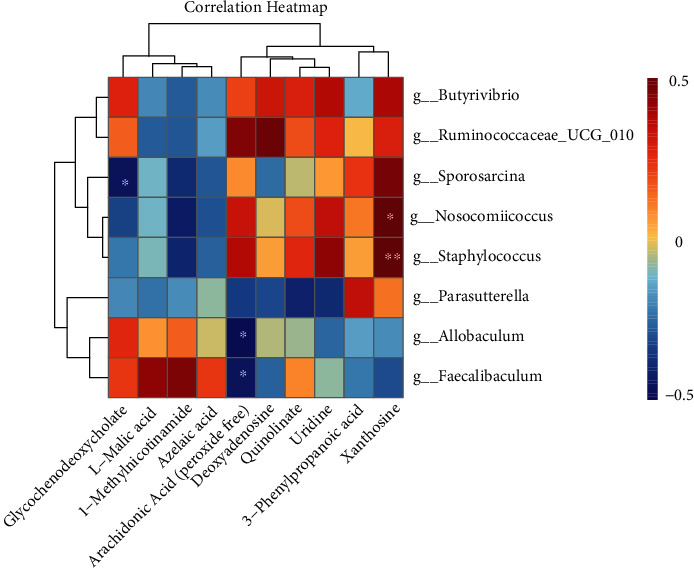
A correlation heat map is used to represent relevance analysis between gut microbiota and fecal biomarkers.

Pearson correlation analysis was used to explore the potential association between gut microbiota genera and fecal biomarkers. The correlation between the two is presented the heat map plot in Figure 12. The results showed that g__*Allobaculum* and g__*Faecalibaculum* were negatively correlated with arachidonic acid (peroxide-free), g__*Sporosarcina* was negatively correlated with glycochenodeoxycholate, and g__*Nosocomiicoccus* and g__*Staphylococcus* were positively correlated with xanthosine.

## 4. Discussion

STC is a common form of functional constipation, characterized by slow colonic motility and delayed elimination of feces [23]. TCM has been used to prevent and treat various diseases for thousands of years, which is characterized by multicomponents, multitargets, personalized, and holistic therapeutic strategies [24, 25]. ZS and BZ are two classical aromatic herbs commonly prescribed for laxative purposes, and most studies have focused on the efficacy of oral administration, while the role of ZBVO in modulating STC and its mechanism are still unclear. In this study, the effect of ZBVO in regulating STC and its mechanism were investigated by transdermal administration at the Shenque acupoint. 16S rRNA gene sequencing and fecal metabolomics were also used to elucidate the mechanism of action of ZBVO in laxatives. Our study showed that ZBVO has therapeutic effects in STC rats, and the main mechanisms are anti-inflammatory, regulation of gastrointestinal hormones, regulation of brain and intestinal peptides, and regulation of intestinal microbiota and endogenous metabolites. ZBVO improved the typical symptoms of STC induced by compound diphenoxylate tablets, especially in the MD and HD groups.

It is hypothesized that the intestinal motility-promoting effect of ZBVO is related to its chemical composition and activity. The highest levels of the chemical components D-limonene, beta-myrcene, and gamma-terpinene found in *Citrus aurantium* volatile oil are associated with the treatment of STC. D-Limonene causes excitation of the smooth muscle in the gastrointestinal tract and regulates immunity [26]. It also has anti-inflammatory and antiseptic properties and can reduce gastric pH and increase the effect of gastric mucus [27, 28]. Zheng et al. [29] found that D-limonene significantly promoted gastric emptying and intestinal peristalsis and inhibited gastrointestinal smooth muscle contraction. The volatile oil of ZS is predicted to exert its pharmacological effects mainly through acetylcholinesterase (ACHE), prostaglandin-endoperoxide synthase 2 (PTGS2), norepinephrine transporter gene (SLC6A2), peroxisome proliferator-activated receptor (PPARA), cholinergic muscarinic 2 (CHRM2), and other related target proteins. It is mainly related to neuroactive ligand-receptor interaction, endocrine resistance, serotonergic synapse, cyclic adenosine monophosphate (cAMP), calcium signaling pathway, and so on [30]. To a certain extent, it confirms the reliability of the ZBVO study for the treatment of STC. Here, the chemical composition of ZBVO was analyzed by GC-MS, and 38 chemical components were identified, among which D-limonene (42.72%), atractylon (27.51%), and *γ*-elemene (7.83%) were identified as the three most abundant chemical components.

Gastrointestinal hormones regulate the movement, secretion, and absorption of the digestive system through different signaling pathways [31]. GAS is mainly secreted by the mucosa of the gastric sinus, duodenum, and small intestine [32]. SP is an endogenous peptide involved in immune regulation and cell proliferation, which has a facilitative effect in the contraction of intestinal smooth muscle and promotes gastrointestinal motility by stimulating the secretion of water and electrolytes from the small intestine and colonic mucosa. Additionally, it regulates the inflammatory response [33]. Some studies have shown that GAS and SP levels were significantly lower in patients with constipation [34]. In this study, the ZBVO administration and PC groups were able to increase the levels of GAS and SP in a dose-dependent manner. Therefore, ZBVO has a laxative effect and promotes intestinal motility after transdermal administration.

Current research in the field suggests that the pathogenesis of STC is closely related to disorders of the brain-gut axis [35]. 5-HT and VIP are expressed in both colonic and hippocampal tissues of animals [36]. 5-HT activates afferent nerve fibers in the intestinal mucosa to regulate local excitation or inhibition, thereby promoting colonic motility and transmission [37]. Bassotti et al. [38] found that the level of 5-HT 4 receptors in STC patients showed a positive correlation with colonic transport capacity, suggesting that the transport system of the colon is closely related to 5-HT. 5-HT is a brain intestinal peptide that binds to the 5-hydroxytryptamine transporter receptor (SERT) and acts on gastrointestinal motility, and some studies have reported that dysbiosis of the intestinal microbiota can have an effect on the expression level of SERT [39]. *Clostridium prazmowski* and *Escherichia coli* secretion products can exert a constraining effect on the synthesis rate of 5-HT, thus affecting the movement of smooth muscle in the gastrointestinal tract [40]. VIP is composed of 28 amino acids, which can relax the smooth muscle of the gastrointestinal tract, thereby slowing down intestinal peristalsis and intestinal dynamics, making defecation difficult and eventually causing constipation. Clinical trials indicate that patients with constipation have lower levels of VIP expression than normal [41]. In the present study, we found that ZBVO could upregulate 5-HT in colonic and hippocampal tissues, while downregulating VIP expression, together exerting a regulatory effect on STC.

Dysbiosis of the intestinal microbiota is closely associated with the development of several pathogenic conditions, such as obesity, gastrointestinal disorders, diabetes, and Alzheimer's disease [42, 43]. In the present study, high-throughput sequencing of the 16S rRNA gene V4 region of the intestinal microbiota showed that the intestinal microbiota of rats induced by diphenoxylate tablets differed significantly from that of normal rats in terms of diversity and composition. And the abundance of *Firmicutes* was increased in the intestine of rats induced by compound diphenoxylate tablets compared with the BC group, but the abundance of the *Bacteroidetes* and *Proteobacteria* was lower than that of the BC group, and the community composition was changed by reducing *Romboutsia* bacteria as well as increasing *Proteobacteria*, *Allobaculum*, and *Ruminococcaceae* after ZBVO administration. To further understand how volatile oils improve constipation in SD rats, the genus level was explored at the level of *in vivo* microbiota. IL-6 and TNF-*α* are closely related to the development of STC [21]. The present study found that ZBVO significantly reduced the serum levels of IL-6 and TNF-*α* and moderately slowed down the inflammatory response. Moreover, *Lactobacillus* and *Blautia* showed a weak positive correlation with TNF-*α*, *Ruminococcaceae* showed a negative correlation with IL-6 and TNF-*α*, and *Blautia* showed a positive correlation with IL-6. There was also a significant positive correlation found between IL-6 and TNF-*α*, suggesting that the change of intestinal microbiota is involved in the regulation of inflammatory factor expression, and the joint action of both contributes to the development and treatment of STC.

Fecal metabolomics indicates that the MC group is clearly separated from the BC group, while the HD group was close to the BC group and showed partial overlap, indicating that metabolite levels *in vivo* had changed significantly compared with the BC group, and after administration of ZBVO, there was a tendency to return to normal performance levels. A total of 30 potential biomarkers associated with STC were identified, and ZBVO intervention significantly regressed 10 of them. Metabolic pathway enrichment analysis revealed that ZBVO may regulate metabolic pathways such as nicotinate and nicotinamide metabolism, purine metabolism, citrate cycle (TCA cycle), pyruvate metabolism, arachidonic acid metabolism, pyrimidine metabolism, and primary bile acid biosynthesis.

Arachidonic acid is an essential fatty acid in the body and is also a precursor to enzymatic and nonenzymatic oxidation products such as prostaglandins, thromboxanes, leukotrienes, lipoxygenins, and isoprostane, which can exert oxidative stress signals associated with inflammation [44]. Studies have shown that after the action of 5-lipoxygenase, arachidonic acid can be converted into leukotriene A4, which is catalyzed by hydrolase and has an inhibitory effect on the immune response and the formation of lymphokines [45]. In addition, studies have shown that epoxyeicosatrienoic acid produced from arachidonic acid can exert anti-inflammatory effects by inhibiting the release of inflammatory factors such as IL-6, TNF-*α*, and IL-1*β* [46]. In this study, arachidonic acid was significantly higher in the ZBVO administration group compared to the MC group, suggesting that ZBVO may exert a regulatory effect on STC by modulating arachidonic acid metabolism to enhance anti-inflammatory effects *in vivo*.

Purine metabolism, TCA cycle, and pyruvate metabolism are all closely related to energy metabolism. Increased energy metabolism in the rat intestine plays an important role in regulating STC, as the peristalsis of the intestine requires a large amount of energy. Purines are among the most abundant metabolites in mammalian cells, and in addition to producing DNA and RNA molecules, purine nucleotides such as adenosine 5′ triphosphate (TP) and guanosine 5′ triphosphate (GTP) are critical for providing cellular energy and intracellular signals, respectively [47]. In this experiment, the loss of appetite and sluggish locomotion in STC rats may be related to the decrease of xanthine nucleosides and thus the decreased energy metabolism, meaning the purine-related xanthine nucleoside is in a disorder in STC rats. After ZBVO regulation, xanthine nucleoside was backregulated, which promoted the recovery of purine metabolism disorder. The citrate cycle is the final metabolic pathway for three major nutrients (amino acids, sugars, and lipids) and is the hub of energy metabolism. L-Malate is an intermediate in the tricarboxylic acid cycle and pyruvate metabolism. In this study, the level of L-malic acid in the MC group was reduced, suggesting that their energy metabolism was disturbed. While ZBVO regulated the TCA cycle, the pyruvate metabolism pathway increased L-malic acid in STC rats, thus providing sufficient energy for intestinal motility. Bile acid has a regulatory effect on gastrointestinal motility and promotes the digestion and absorption of lipids in food [48]. In the case of gastrointestinal motility disorders, such as STC, the hepatic and intestinal circulation of bile acids is affected. Arachidonic acid is a product of cholesterol in the presence of 7*α* hydroxylase, and here, the level of glycocholic acid in the feces in the MC group was significantly reduced, suggesting that primary bile acid biosynthesis was disrupted by the diphenoxylate tablets. In contrast, the administration of ZBVO resulted in a significant increase in arachidonic acid, suggesting that ZBVO regulates primary bile acid biosynthesis, which may explain the rheumatological, spleen-building, and digestive effects of ZBVO.

## 5. Conclusion

This study demonstrated the alleviating effect of transdermal administration of ZBVO on constipation in STC rats. ZBVO can promote intestinal peristalsis, increase fecal water content, regulate gastrointestinal hormone level (GAS and SP), reduce the inflammatory response (IL-6 and TNF-*α*), and regulate brain-intestinal peptides (5-HT and VIP) after transdermal administration. In addition, the composition of the intestinal microbiota is improved by ZBVO's reduction of harmful bacteria and promotion of beneficial bacteria. This study may provide efficacious drugs for STC patients. This study explores a new drug delivery component and drug delivery route for ZS-BZ-regulated STC and provides a new direction for the development of aromatic drug macrohealth products.

## Figures and Tables

**Table 1:** Main chemical composition and percentage of ZBVO.

No.	Retention time (min)	Compound	Chemical formula	Relative amount (%)	CAS
1	7.337	*β*-Pinene	C_10_H_16_	1.18	000127-91-3
2	7.520	*β*-Myrcene	C_10_H_16_	0.32	000123-35-3
3	8.280	D-Limonene	C_10_H_16_	42.72	005989-27-5
4	8.470	*β*-Ocimene	C_10_H_16_	0.55	013877-91-3
5	8.912	Linalyl oxide	C_10_H_18_O_2_	0.19	005989-33-3
6	9.170	Terpinolene	C_10_H_16_	0.17	000586-62-9
7	9.333	Linalool	C_10_H_18_O	1.34	000078-70-6
8	10.616	Terpinen-4-ol	C_10_H_18_O	0.39	000562-74-3
9	10.819	(-)-*β*-Fenchyl alcohol	C_10_H_18_O	0.45	000470-08-6
10	11.240	(-)-trans-Carveol	C_10_H_16_O	0.35	001197-07-5
11	11.417	Carveol	C_10_H_16_O	0.17	000099-48-9
12	11.620	d-Carvone	C_10_H_14_O	0.15	002244-16-8
13	12.645	Dodecamethylcyclohexasiloxane	C_12_H_36_O_6_Si_6_	0.14	000540-97-6
14	12.978	*α*-Terpinene	C_15_H_24_	0.38	000099-86-5
15	13.752	*β*-Elemene	C_15_H_24_	0.52	000515-13-9
16	13.942	(-)-Cyperene	C_15_H_24_	0.17	002387-78-2
17	14.193	*β*-Caryophyllene	C_15_H_24_	1.08	000087-44-5
18	14.336	*γ*-Elemene	C_15_H_24_	7.83	029873-99-2
19	14.655	Humulene	C_15_H_24_	0.47	006753-98-6
20	14.899	*γ*-Cadinene	C_15_H_24_	0.32	030021-74-0
21	15.008	Germacrene D	C_15_H_24_	2.05	023986-74-5
22	15.089	*β*-Selinene	C_15_H_24_	1.34	017066-67-0
23	15.184	Valencene	C_15_H_24_	0.32	004630-07-3
24	15.483	(+)-delta-Cadinene	C_15_H_24_	0.62	000483-76-1
25	15.714	Isoledene	C_15_H_24_	3.71	095910-36-4
26	15.802	3-(2′,4′-Dimethylpenta-1′,4′-dien-3′-ylidene)-6,6-dimethylcyclohex-1-ene	—	0.76	997214-07-9
27	15.890	Nerolidol	C_15_H_26_O	0.20	007212-44-4
28	16.067	Isolongifolene, 9,10-dehydro-	C_15_H_22_	0.85	997214-17-0
29	16.630	4-Bromo-1-naphthylamine	C_10_H_8_BrN	0.23	002298-07-9
30	16.841	Spathulenol	C_15_H_24_O	1.01	006750-60-3
31	17.296	Atractylon	C_15_H_20_O	27.51	006989-21-5
32	17.526	Vulgarol B	C_20_H_36_O_2_	0.22	011056-03-4
33	17.662	(1R,4Ar,8aR)-1,4a-Dimethyl-7-propan-2-ylidene-3,4,5,6,8,8a-hexahydro-2H-naphthalen-1-ol	C_15_H_26_O	0.15	000473-04-1
34	17.839	1-Hydroxy-1-methyl-7(methylethenyl)[1,2,3,3a,4,5,6,7]octahydro azulene	—	0.17	997228-20-5
35	18.083	4-Methoxy-5-oxidanyl-naphthalene-2-carboxylic acid	—	0.72	997266-09-1
36	19.889	1,5,9-Trimethyl-2-oxatricyclo[7.3.0.0(3,8)]dodec-3(8),4,6-triene	—	0.82	997213-83-9
37	20.106	Palmitic acid	C_16_H_32_O_2_	0.30	000057-10-3
38	20.737	3-Phenyl-1,2-dihydrocyclopenta[a]indene	C_18_H_14_	0.16	997312-45-2

**Table 2:** Potential biomarkers in rat feces after modeling (*n* = 6).

Name	Formula	Detected *m*/*z*	VIP value	MC/BC	HMDB ID	KEGG
Protoporphyrin IX	C_34_H_34_N_4_O_4_	563.2598	1.49	↓^#^	HMDB0000241	C02191
Uridine	C_9_H_12_N_2_O_6_	243.0648	2.09	↓^##^	HMDB0000296	C00299
Nervonic acid	C_24_H_46_O_2_	365.3481	1.63	↓^#^	HMDB0002368	C08323
Cer(d18:1/18:1(9Z))	C_36_H_69_NO_3_	564.5288	1.83	↓^##^	HMDB0004948	C00195
Lathosterol	C_27_H_46_O	369.3463	1.43	↓^#^	HMDB0001170	C01189
Lanosterol	C_30_H_50_O	409.3744	1.75	↓^#^	HMDB0001251	C01724
Myristoleic acid	C_14_H_26_O_2_	191.1767	1.70	↓^#^	HMDB0002000	C08322
N-Glycolylneuraminic acid	C_11_H_19_NO_10_	326.1047	1.46	↓^#^	HMDB0000833	C03410
Glycochenodeoxycholate	C_26_H_43_NO_5_	432.3012	1.83	↓^##^	HMDB0000637	C05466
Arachidonic acid (peroxide-free)	C_20_H_32_O_2_	269.2062	1.85	↓^##^	HMDB0001043	C00219
Phytanic acid	C_20_H_40_O_2_	354.3308	1.52	↓^#^	HMDB0000801	C01607
L-Glutamine	C_5_H_10_N_2_O_3_	145.0521	1.63	↓^#^	HMDB0000641	C00064
Uracil	C_4_H_6_N_2_O_2_	113.0326	1.51	↓^#^	HMDB0000300	C00106
Quinolinate	C_7_H_5_NO_4_	166.0165	1.62	↓^#^	HMDB0000232	C03722
Alpha-D-glucose	C_6_H_12_O_6_	179.0571	1.57	↓^#^	HMDB0003345	C00267
L-Serine	C_3_H_7_NO_3_	104.0343	1.44	↓^#^	HMDB0000187	C00065
1,7-Dimethylxanthine	C_7_H_8_N_4_O_2_	180.0554	1.84	↓^##^	HMDB0001860	C13747
D-Threitol	C_4_H_10_O_4_	143.0353	1.63	↓^#^	HMDB0004136	C16884
*trans*-Dehydroandrosterone	C_19_H_28_O_2_	287.1868	1.56	↓^#^	HMDB0000077	C01227
1-Methylnicotinamide	C_7_H_9_N_2_O	138.0576	2.16	↓^##^	HMDB0000699	C02918
L-Asparagine	C_4_H_8_N_2_O_3_	113.0240	1.51	↓^#^	HMDB0000168	C00152
Xanthosine	C_10_H_12_N_4_O_6_	285.0836	1.98	↓^##^	HMDB0000299	C01762
D-Sorbitol	C_6_H_14_O_6_	163.0621	1.44	↓^#^	HMDB0000247	C00794
4-Hydroxycinnamic acid	C_9_H_8_O_3_	147.0415	1.51	↓^#^	HMDB0002035	C00811
L-Malic acid	C_4_H_6_O_5_	133.0146	1.52	↓^#^	HMDB0000156	C00149
3-Phenylpropanoic acid	C_9_H_10_O_2_	149.0622	1.50	↑^#^	HMDB0000764	C05629
Aminopterin	C_19_H_20_N_8_O_5_	439.1542	1.95	↑^##^	HMDB0001833	D02527
Azelaic acid	C_9_H_16_O_4_	187.0997	2.05	↑^##^	HMDB0000784	C08261
Deoxyadenosine	C_10_H_13_N_5_O_3_	252.1042	1.62	↑^#^	HMDB0000101	C00559
1-Methylhistamine	C_6_H_11_N_3_	112.0843	1.64	↑^#^	HMDB0000898	C05127

MC/BC: comparison between the model control group and the blank control group, ^#^*p* < 0.05 and ^##^*p* < 0.01; “↓” or “↑” compared with the blank control group, the metabolites in the model control group decreased or increased significantly.

**Table 3:** Differential metabolites in rat feces after administration (*n* = 6).

Name	Formula	Detected *m*/*z*	VIP value (MD/MC)	VIP value (HD/MC)	HMDB ID	KEGG
Uridine	C_9_H_12_N_2_O_6_	243.0648	1.54 ↑^#^	—	HMDB0000296	C00299
Glycochenodeoxycholate	C_26_H_43_NO_5_	432.3012	1.47 ↑^#^	—	HMDB0000637	C05466
Arachidonic acid (peroxide-free)	C_20_H_32_O_2_	269.2062	1.54 ↑^#^	—	HMDB0001043	C00219
Quinolinate	C_7_H_5_NO_4_	166.0165	1.69 ↑^##^	1.75 ↑^##^	HMDB0000232	C03722
1-Methylnicotinamide	C_7_H_9_N_2_O	138.0576	1.45 ↑^#^	—	HMDB0000699	C02918
Xanthosine	C_10_H_12_N_4_O_6_	285.0836	1.48 ↑^#^	1.91 ↑^##^	HMDB0000299	C01762
3-Phenylpropanoic acid	C_9_H_10_O_2_	149.0622	1.81 ↓^##^	—	HMDB0000764	C05629
Azelaic acid	C_9_H_16_O_4_	187.0997	1.78 ↓^##^	—	HMDB0000784	C08261
L-Malic acid	C_4_H_6_O_5_	133.0146	—	1.79 ↑^##^	HMDB0000156	C00149
Deoxyadenosine	C_10_H_13_N_5_O_3_	252.1042	—	1.48 ↓^#^	HMDB0000101	C00559

Note: “↓” or “↑” compared with the model control group, the metabolites in the middle-dose (MD) group and the high-dose (HD) group decreased or increased significantly.

## Data Availability

All data used to support the findings of this study are available from the corresponding author upon request.

## Supplementary Materials

Supplementary Materials Supplementary Figure 1: plot of sample dilution curves and Shannon-Wiener curves for operable taxonomic units in the population. Supplemental Table 1: elution gradient.
