# Supplementary material for: Transdermal Administration of Volatile Oil from Citrus aurantium-Rhizoma Atractylodis Macrocephalae Alleviates Constipation in Rats by Altering Host Metabolome and Intestinal Microbiota Composition
Source: Oxid Med Cell Longev. 2022 Jan 18;2022:9965334. doi: 10.1155/2022/9965334 (PMC8789429; doi:10.1155/2022/9965334)
Supplement: Supplementary Materials — Supplementary Figure 1: plot of sample dilution curves and Shannon-Wiener curves for operable taxonomic units in the population. Supplemental Table 1: elution gradient. [file 9965334.f1.docx]

Supplementary Figure 1 Plot of sample dilution curves and Shannon-Wiener curves for operable taxonomic units in the population. Supplemental Table 1 Elution gradient.
